# Experiences with remote ethics consultation: a qualitative study with ethics consultants in Germany

**DOI:** 10.1186/s12910-026-01401-x

**Published:** 2026-02-10

**Authors:** Esther Braun, Florian Funer, Ruben A. Sakowsky, Robert Ranisch, Joschka Haltaufderheide

**Affiliations:** 1 Junior Professorship for Medical Ethics with a Focus on Digitization, Faculty of Health Sciences Brandenburg, University of Potsdam, Potsdam, Germany; 2 Institute of Ethics and History of Medicine, Medical Faculty, Eberhard Karls University Tübingen, Tübingen, Germany

**Keywords:** Clinical ethics support, Ethics committees, Moral case deliberation, Digitization, Digital communication, Video conferencing

## Abstract

**Background:**

The use of remote methods such as video conferencing has the potential to improve access to ethics consultations, particularly in outpatient and rural healthcare settings. Although their use has increased since the COVID-19 pandemic, little is known about ethics consultants’ experiences with remote consultations so far.

**Methods:**

We conducted three focus groups with 14 certified ethics consultants in Germany in October 2024 to investigate their experiences with, and attitudes towards, remote ethics consultations. The data was analysed using structuring qualitative content analysis.

**Results:**

Participants reported experiences with a range of remote methods, including email and phone for short consultations, and videoconferencing for full ethics consultations. Videoconferencing was primarily used when in-person meetings were not feasible. Attitudes towards video-based consultations varied, and consultants with more experience with this technology tended to view it more favourably. Reported advantages included improved accessibility, especially in rural areas, easier scheduling, and the ability to involve additional stakeholders. Disadvantages included technical challenges and concerns about privacy. Participants reported that video-based ethics consultations reduce non-verbal communication and emotional expression. According to some, this made video consultations more structured and egalitarian, while others found this disadvantageous. Participants emphasised the need for specific preparation for video-based ethics consultations, such as ensuring adequate technical set-up or assigning roles to monitor emotional dynamics. Possible challenges regarding the involvement of patients and relatives in remote consultations were noted. While some participants viewed remote formats as more suitable for less emotionally intense cases, others saw no inherent limitations regarding consultation topics.

**Conclusion:**

Our findings indicate that ethics consultants regard video-based remote consultations as a viable alternative when in-person meetings are not feasible. The study identified specific communicative challenges that should be addressed in future training for ethics consultants. Further research is needed to ensure that the implementation of remote ethics consultation can realise its advantages and mitigate possible disadvantages. This includes exploring the perspectives of patients and relatives.

**Supplementary Information:**

The online version contains supplementary material available at 10.1186/s12910-026-01401-x.

## Introduction

Clinical ethics consultations provide support in addressing ethical issues in healthcare settings [1, 2]. In hospitals, they are provided by clinical ethics consultants who usually are part of a clinical ethics committee [3]. Healthcare professionals, as well as patients, their relatives, or legal representatives, can contact the clinical ethics committee with requests for support with ethical questions, although requests are most commonly made by healthcare professionals [4, 5]. Such support can take different forms: sometimes, requests can be resolved in short, initial consultations, for example when a healthcare professional contacts the clinical ethics committee with a question that can be answered in a single conversation with a clinical ethics consultant. In contrast, a full clinical ethics consultation consists of a structured discussion with several participants that is moderated by one or several clinical ethics consultants. An ethics consultation can include members of the treatment team, patients, legal representatives, and/or relatives [6]. A variety of methods for conducting ethics consultations have been developed [2]. Common approaches include gathering relevant facts about the case, hearing the perspectives of all involved parties, identifying possible courses of action, and providing recommendations for action based on joint ethical reasoning [1, 2].

In Germany, the focus of this study, clinical ethics committees have been established since the 1990s, and are now present in most hospitals [7–12]. Next to ethics consultations, the tasks of clinical ethics committees include developing institutional ethical guidelines for hospitals, delivering ethics education for staff and advising hospital management on ethical questions at the organisational level [13]. The German association for medical ethics, the Academy of Ethics in Medicine (AEM), sets national standards for ethics consultation and certifies consultants at three competency levels: “Ethics Consultant in Healthcare” (level 1), “Coordinator for Ethics Consultation in Healthcare” (level 2), and “Trainer for Ethics Consultation in Healthcare” (level 3) [5, 14].

More recently, outpatient clinical ethics services have emerged in Germany, which offer ethics consultation outside of hospitals – for example in nursing homes and for general practitioners [15]. The significance of these services was highlighted by a recent statement of the Central Ethics Committee of the German Medical Association, which called for their increased implementation [16]. Unlike hospital-based clinical ethics committees that provide support for one institution, outpatient clinical ethics services are independent entities that provide ethics consultations to various institutions within a region [8]. They follow their own operational procedures and offer advisory support in addressing ethical conflicts concerning patients treated in outpatient settings. Outpatient ethics consultation is not legally regulated and its recommendations are not binding, as the final responsibility for patient care remains with the treatment team – just as in the case of clinical ethics committees within hospitals. However, because outpatient services are external to healthcare institutions, consultations legally require the explicit consent of the patient or their legal representative, or are conducted anonymously – in contrast to hospital settings, where patients typically consent to necessary information being shared across disciplines as part of standard care, which includes ethics consultations [17].

Outpatient ethics consultations particularly often require coordinating participants from diverse groups and settings – such as relatives, general practitioners, nurses, and representatives from nursing homes or palliative care teams. Although ethical conflicts are often time-sensitive, it can be difficult to organise ethics consultations in a time-sensitive manner when it is necessary to coordinate multiple stakeholders across different institutions. These logistical challenges are especially relevant in rural areas or large geographic areas with a low density of healthcare professionals and trained ethicists [15].

Remote formats – that is, consultations via email, telephone, or videoconference – could offer a solution to help address such logistical challenges. A recent survey highlights the demand for increased availability of ethics consultation via telephone among general practitioners in Germany [18]. Next to the outpatient context, remote formats can also be used in inpatient settings. For example, videoconferencing can help provide ethics consultations to hospitals without their own clinical ethics committees, particularly in rural areas [19]. Moreover, clinical ethics committees can use remote formats to involve additional stakeholders in ethics consultations, or to comply with contact restrictions. In Germany, both clinical ethics committees and outpatient clinical ethics services made initial experiences with video-based consultations during the COVID-19 pandemic, although remote formats have not yet been widely implemented.

Generally, the COVID-19 pandemic increased the demand for alternatives to in-person consultations [20], especially video-based formats, across healthcare settings. Initial research has examined the effects of remote consultations in different settings [21], attitudes and acceptance [22], as well as changes in communication between in-person and remote interaction [23, 24].

With respect to remote ethics consultations, first experiences have been reported from the US [25–27]. These initial reports describe how telemedicine can help offer ethics support to hospitals that lack local ethicists, particularly in rural areas. They also indicate challenges, such as technical barriers, concerns about confidentiality, and limitations regarding the quality of communication in remote consultations [25–27]. However, no study to date has explored the perspectives of ethics consultants on the specific challenges of conducting ethics consultations remotely.

Consequently, the goal of this qualitative study was to explore how ethics consultants perceive the opportunities and challenges of remote formats within the German context. By identifying practical benefits and obstacles, our findings aim to inform best practices for remote ethics consultation, guide targeted curricula, and clarify when remote formats are most appropriate.

## Methods

### Study design

We conducted a focus group study with clinical ethics consultants to explore their experiences with, and attitudes towards, remote ethics consultation. We chose a focus group format over individual interviews because it allows participants to interact and build on each other’s ideas. This can generate richer discussions, reveal shared experiences, and highlight areas of disagreement among participants, which is particularly valuable for an underexplored topic. We followed the standards for reporting qualitative research proposed by O’Brien et al. [28].

### Researcher reflexivity

Our research team combined expertise from medical ethics, medicine, philosophy, theology and health economics. Several authors are certified ethics consultants who have experience conducting ethics consultations remotely and were involved in establishing *Ethikberatung Brandenburg*, a remote outpatient clinical ethics consultation service now established at the Junior Professorship for Medical Ethics with a Focus on Digitization, Faculty of Health Sciences Brandenburg, University of Potsdam. This service provides ethics consultations in outpatient settings, as well as for hospitals that lack own ethics consultation services, in the German state of Brandenburg. The team of *Ethikberatung Brandenburg* comprises five members: one ethics consultant certified at level K1, two at level K2, and one at level K3 according to AEM standards, as well as one member who is an experienced medical ethicist without AEM certification. The results of our study were intended to inform the development of this service by identifying challenges and best practices in remote ethics consultation based on the experiences of others, thereby allowing us to integrate these insights into the design and implementation of *Ethikberatung Brandenburg*.

### Participant selection

We sent invitations to participate in the study via email to representatives of organisations providing outpatient clinical ethics consultation services in Germany via a voluntary, publicly available register of outpatient clinical ethics consultation services provided by the AEM (available at https://aem-online.de/ausserklinische-ethikberatung/). In addition, we distributed the study invitation via the AEM’s mailing list, which reaches both inpatient and outpatient ethics consultants in Germany. Inclusion criteria were being a certified ethics consultant in Germany and having either experience with or an interest in conducting video-based clinical ethics consultations in inpatient or outpatient contexts. No additional exclusion criteria were applied. We used purposive sampling, a sampling method in which participants are intentionally selected based on specific characteristics, to ensure diversity of participants with respect to gender, age, level of experience and certification. The number of participants resulted both from participants’ availability and the achievement of thematic saturation after three focus groups.

### Data collection

We conducted three focus groups with 4–6 participants each via videoconference (Zoom) in October 2024. The focus groups lasted between 79 and 94 min (mean: 88 min) and were moderated by EB, JH, and RS using a semi-structured discussion guide (an English translation is provided in the online supplement). While the discussion guide included questions on both outpatient clinical ethics services and remote ethics consultation, this manuscript focuses exclusively on the latter topic. We created audio recordings of the focus groups, which were initially transcribed with the software noScribe. A student assistant checked the initial transcription against the audio recording and corrected the transcripts, and EB pseudonymised the transcripts.

### Data analysis

The data were analysed with the software MAXQDA 2024 following the principles of structuring qualitative content analysis according to Kuckartz [29]. The coding system was developed both deductively, based on the discussion guide, and inductively, based on topics that emerged during the focus group discussions. First, meaning units (i.e., segments of text that relate to a specific meaning relevant to the research question) were identified and assigned initial codes. Then, similar codes were grouped into subcategories, which were abstracted into broader categories (themes). The process was iterative, with constant comparison between data, codes, and themes. All transcripts were coded by EB and one other researcher (FF or JH) to ensure consistency and reliability. Coding discrepancies were discussed in team meetings among all authors until consensus was reached.

### Ethical considerations

All participants received written information about the study via email at least 24 h before the focus groups. This information described the study’s aim, the type of data collected, procedures for anonymisation, data access, and secure storage. Participants were invited to contact the research team with questions prior to participation. At the beginning of each focus group, the study’s purpose was reiterated verbally, and participants again had the opportunity to ask questions. Verbal consent was obtained before the discussion began. Written informed consent was collected in advance of participation through signed consent forms returned to the research team via email. Ethical approval for the study was obtained from the Research Ethics Committee of the University of Potsdam (registration number 69/2024).

## Results

A total of 14 ethics consultants participated in our study. The sociodemographic characteristics of focus group participants are presented in Table 1.

**Table 1:** Characteristics of focus group participants

Dimension		Description
Number of participants		14
Mean age in years (range)		52.9 (30–69)
Gender		
	Female	7
	Male	7
Mean experience with conducting clinical ethics consultations in years (range)		10.3 (1–27)
Certification as an ethics consultant according to the standards of the German Academy of Ethics in Medicine		
	Level K1	5
	Level K2	4
	Level K3	5

Five themes emerged from our analysis: (1) use and functions of different remote formats, (2) attitudes towards remote ethics consultation using videoconferencing, (3) advantages of video-based ethics consultations, (4) disadvantages of video-based consultations, and (5) differences between in-person and video-based ethics consultations. All themes and their respective subcategories are presented in Table 2. In the following, we describe these themes and present representative quotes from the focus group discussions, which were translated from German to English by EB.

**Table 2:** Themes and subcategories

Theme	Subcategory
Use and functions of different remote formats	
	Email-based communication for clarification and preparation
	Telephone-based initial consultations or resolution
	Videoconference-based full consultations
Attitudes towards remote ethics consultation using videoconferencing	
	Confidence based on prior experience
	Reservations linked to limited experience
	Overall preference for in-person consultations
Advantages of video-based consultations	
	Bridging geographical distances
	Inclusion of additional participants
	Easier scheduling
	Infection prevention
Disadvantages of video-based consultations	
	Technical aspects
	Privacy concerns
	Lack of physical proximity
Differences between in-person and video-based ethics consultations	
	Preparation
	Communication during consultations
	Follow-up and debriefing
	Including patients and relatives
	Appropriate types of requests

### Use and functions of different remote formats

Participating ethics consultants reported on experiences with different remote formats using email, telephone, and videoconferencing.

Participants reported using email mainly to clarify and document requests before a consultation, asking that cases be described in writing so that they had concrete information to work with in subsequent discussions.

Participants stated that they frequently conducted short initial consultations via telephone, which was described as the most commonly used remote format. These short consultations often resolved the initial question, so that a full ethics consultation did not become necessary:

*And then the initial contact is made by telephone. And as I said*,* our experience is that approximately half of the requests are ultimately resolved with this initial contact*,* with the telephone call. It’s possible that you have an initial contact like this*,* discuss it in the phone call*,* and that then maybe a week later you get another response*,* but now a case consultation would be desirable or useful. But in many cases*,* it is already resolved with a telephone consultation. (S02*,* focus group 1)*

Video conferencing was used to conduct full ethics consultations by ethics consultants in hospitals as well as in outpatient settings. Some participants gained initial experiences with video-based consultations during the COVID-19 pandemic, as these became necessary due to contact restrictions, while others first began to use such formats independently of the pandemic. Regarding technical requirements for video conferencing, study participants reported challenges with hybrid setups, especially when multiple participants used one device. In contrast, video conferences with small groups, where each person joined from their own device, was generally perceived as more effective and technically reliable.

The following themes focus on study participants’ experiences with conducting full ethics consultations via videoconferencing, which we also refer to as “video consultations”.

### Attitudes towards remote ethics consultations using videoconferencing

Participants expressed mixed attitudes towards video consultations. Analysis of the qualitative data indicated a tendency for focus group participants who were more experienced with remote formats to have a more positive attitude, while less experienced participants were more sceptical and had doubts about whether conducting ethics consultations via videoconferencing was feasible, for example due to technical challenges. For instance, a participant who had not used video consultations before expressed a sceptical attitude:

*Now we lack experience here a bit*,* but what was mentioned earlier*,* so*,* to what extent can it be used*,* can it be operated*,* are the conditions in place*,* is there a stable internet connection*,* so*,* these are things that cannot be taken for granted and that require a lot of clarification. So I see that rather critically. (S06*,* focus group 2)*

On the other hand, a participant who had been using video consultations regularly stated:

*And so*,* it’s a tool that works well. If people are reasonably tech-savvy*,* it’s not a problem at all […] so there’s no big difference compared to an in-person consultation. (S09*,* focus group 3)*

Overall, most participants in our study preferred in-person consultations and used video consultations as a substitute when face-to-face meetings were not possible. Some did not regard video consultations as an adequate replacement for an in-person meeting, instead viewing them as a useful option for preparation ahead of a complete ethics consultation.

### Advantages of video-based ethics consultations

Participants particularly valued the ability of video-based ethics consultations to bridge geographical distances, making them especially suitable for rural settings. They allow including family members who live far away and would otherwise not be able to participate in ethics consultations, as well as professionals from other institutions. This was particularly important for members of outpatient ethics consultation services, allowing them to take on requests from individuals who lived further away. Participants also found ethics consultations via videoconference less challenging to schedule than in-person meetings, which allowed finding appointments faster, and reported that this was also valued by clinicians who request ethics consultations:

*In our case*,* online consultations are actually often requested. So it’s not the case that we only use it when somehow nothing else is possible. Because it’s quicker*,* because it’s more convenient for everyone. Because the doctors in their practices and the person in the care home and the care team can simply join in. And sometimes it’s just the time*,* so everyone would have to wait for it longer. That’s why it’s just*,* everyone is happy that they can now*,* that they can quickly get a solution to their problem. (S03*,* focus group 1)*

Participants also found video consultations particularly valuable during the COVID-19 pandemic, as they allowed ethics consultants to adhere to infection prevention guidelines.

### Disadvantages of video-based ethics consultations

Some participants regarded the technical aspects of video-based ethics consultations as disadvantageous, partly because consultants themselves or participants in consultations lacked “technological aptitude”, which was also described as an age-related issue. Some regarded the technical prerequisites needed for video consultations as a disadvantage, particularly in rural areas in Germany, where internet connection is often unstable. This was described as particularly unfavourable given that remote consultations offer the greatest benefits in rural locations:

*[W]e have the greatest need in rural areas*,* but the fewest possibilities. Because that’s where the biggest problems are with the technology*,* with the connection*,* so the cat is chasing its own tail. (S06*,* focus group 2)*

Some participants expressed concerns about privacy and data protection, which led one ethics consultant to avoid using video-based consultations altogether. Another disadvantage was the lack of physical proximity and possibility for “touch”, which some participants regarded as particularly important for people who request ethics consultations:

*​​We are professional groups […] where everything is also about touch. That means you have to be touched and also touch the person that it’s about. And the relatives*,* too. That means either direct physical touch or allowing yourself to be touched in a kind of closeness that cannot be realised in the same way with remote techniques. (S08*,* focus group 2)*

### Differences between in-person and video-based ethics consultations

Participants described various changes and particularities that they had experienced as a result of conducting ethics consultation via videoconference compared to in-person consultations. Our analysis identified that these referred to several stages and aspects of planning and conducting an ethics consultation, which together formed the theme “Differences between in-person and video-based ethics consultations”. This theme comprised five subcategories: preparation, communication during consultations, follow-up and debriefing, including patients and relatives, and appropriate types of requests. The following sections describe these subcategories in more detail

#### Preparation

Focus group participants noted that video consultations required changes in preparing for an ethics consultation compared to in-person consultations. First, it was necessary to make sure that technical requirements were met: for example, each participant had to access the meeting from their own device in a quiet environment. Some ethics consultants reported providing a phone number that participants could call in case of technical difficulties.

Moreover, it was noted that changes in video-based communication required specific preparation, for example, by assigning moderators to have an eye on emotional dynamics in specific participants:

*As with online*,* a certain awareness of emotional changes in the people involved can possibly be lost*,* we have always agreed in advance who will keep an eye on which people in order to be aware of any changes in mood as far as possible*,* so that we can deal with them accordingly*,* recognise emotional overwhelm at an early stage and prevent it from occurring in the first place. So I think*,* this mentorship*,* which we are also familiar with from in-person consultations*,* where the ethics team thinks about who will keep half an eye on which person*,* is even better in the digital format. (S12*,* focus group 3)*

#### Communication during consultations

Participants noted that non-verbal communication was largely lost in video consultations. For example, in-person consultations allowed them to register whether a participant was nervous or aggressive by observing how they entered a room, greeted someone, or sat in a chair, which was not possible during video consultations. They noted that emotional dynamics were less noticeable in video consultations:

*And also*,* when the mood somehow starts to change*,* I notice that much better in person than when I do it online. (S10*,* focus group 3)*

Some found that this made it more difficult to moderate video-based ethics consultations because it was easier to overlook certain aspects, such as a participant rolling their eyes. It was also noted that video consultations lowered the threshold for exiting a conversation, for example, by answering a phone call.

Some participants, however, characterised the reduced ability to convey emotions in video consultations as a benefit. They noted that a lesser degree of emotionality allowed for more “structured” consultations, and could protect participants from hierarchical and aggressive behaviour, for example, by senior hospital staff.

By limiting each participant in an ethics consultation to their own “video square”, online consultations were regarded as promoting egalitarian communication:

*Now that (S11) has also said it*,* that everyone is actually very limited by their [video] square and somehow*,* everyone is practically standing next to each other*,* too*,* this may even have a more empowering effect*,* at least for some relatives and patients*,* that you don’t sit next to each other in this digital setting*,* so to speak*,* but rather that everyone gets their own protected space*,* but then very decidedly gets their own time. (S14*,* focus group 3)*

Overall, ethics consultants found that they had to be more focused when moderating video-based consultations, for example, by checking that each participant had the ability to contribute to the conversation. However, this could also make ethics consultations more structured, particularly because the online format allowed moderators to have an eye on all individual participants:

*But I think it works very*,* very well online*,* too*,* because in this Zoom format with these squares and so on*,* you really have all the participants in your view. Sometimes even better than with in-person consultations. Because*,* we sometimes have consultations in some kind of meeting room in the hospital. There you’ve got very long*,* narrow tables. Then it’s often difficult to keep an eye on all participants. (S02*,* focus group 1)*

#### Follow-up and debriefing

Ethics consultants noted that informal opportunities for debriefing and follow-up, such as brief conversations while saying goodbye to participants in the hallway after an in-person consultation, were lost in video-based consultations. They stated that this posed the risk of leaving participants to process the emotional impact of the ethics consultation on their own:

*Yes*,* the resonance is simply lacking. You don’t know what the people affected or the people who are present*,* how they will feel after the entire consultation. That is difficult to sense. Even if you have the feeling*,* oh dear*,* now we’re ending the entire session*,* and you have the feeling that you can’t really let them go home*,* but still*,* I don’t know. (S01*,* focus group 1)*

Having the opportunity to debrief was also described as psychologically important for ethics consultants themselves, particularly after difficult case consultations. The ethics consultants therefore noted that follow-up meetings with participants and among themselves were particularly important in online settings. This could be realised via telephone or video conferencing.

#### Including patients and relatives

While remote ethics consultations have the advantage of potentially including relatives who live further away, several participants found it challenging to involve patients or their relatives in video consultations. Some found the online format inappropriate for addressing complex, emotionally challenging questions that deeply affect patients and their relatives, who often are experiencing personal crises. They noted that the lack of informal follow-up after a remote ethics consultation particularly affected these groups:

*But if*,* for example*,* relatives are involved*,* for whom the situation has a completely different meaning*,* then I would always try to make use of an in-person meeting. I would also find it difficult*,* myself as a relative*,* if it was suggested to me that we’re negotiating*,* I’m going to put it a bit bluntly*,* we’re negotiating about the future of your mother or father today and we’ll do that here with these funny little colourful squares. And at some point the screen goes black and then I’m sitting there alone with this decision. I couldn’t take responsibility for that either*,* as a moderator*,* for example. (S06*,* focus group 2)*

Some participants, however, found involving patients and relatives in ethics consultations challenging in general, independently of whether a consultation took place remotely or in person. They noted that successfully including patients in ethics consultations may require additional support, particularly for patients with disabilities. Yet, participants stressed that involving patients and relatives in ethics consultations is often essential to understand their perspectives and to gain a more complete understanding of the respective ethical issue.

#### Appropriate types of requests

Some participants stated that remote formats were less appropriate for emotionally challenging consultations that have potentially serious consequences for patients or relatives, for example, in cases where patients had a wish for death, or when members of the treatment team were highly emotionally involved in the case. Some participants believed that concrete and urgent decisions should only be made in person because they are often emotionally charged.

Some viewed remote ethics consultations as particularly appropriate for preventative ethics consultations, training settings, consultations with participants the moderators already knew, or “factual” discussions that involved a lesser degree of emotionality.

Other participants, however, especially those who were more experienced with remote consultations, did not regard any topics as particularly suitable or unsuitable for video-based consultations.

## Discussion

### Attitudes and experiences

In this study, we identified a range of experiences with, and attitudes towards, remote ethics consultations among ethics consultants in Germany. Participants reported using several remote technologies, including email, telephone and videoconferencing. Participants reported that telephone consultations could often quickly resolve ethical questions, so that full consultations did not become necessary. They reported using videoconferencing for full ethics consultations in inpatient and outpatient settings, typically when in-person meetings were not feasible. The qualitative analysis indicated a trend whereby attitudes towards video-based consultations appeared to correlate with participants’ experience with this technology: those who had more experience using videoconferencing for ethics consultation tended to evaluate it more positively. This could suggest that familiarisation to remote formats reduces initial scepticism. This is in line with research on remote consultations in other medical settings, which shows effects of familiarisation, increased acceptance and confidence through use [30, 31].

Yet, it is also possible that consultants who already have positive attitudes towards such formats use them more often, while those with negative attitudes avoid them. Future research should examine this bidirectional relationship more systematically to determine whether training and usage alone can shift attitudes or if deeper professional values and expectations drive preferences towards remote formats.

### Advantages and disadvantages

Participants identified several benefits of video-based consultations, including greater geographical reach, increased accessibility – especially in underserved rural areas –, the ability to include relatives who live far away and professionals from different institutions, and easier scheduling. These advantages align with broader arguments for the utility of telehealth consultations [24, 32].

Disadvantages and points of scepticism included technical difficulties and challenges due to limited technological literacy. Notably, the areas most in need of remote formats – those with limited access to in-person ethics consultation – are often the very regions where internet infrastructure and technological competence are the weakest. This represents a structural challenge to equitable implementation.

### Involvement of patients and relatives

A similar challenge emerged with respect to the involvement of patients and relatives in remote ethics consultations: while one important advantage of remote consultations is the possibility of including relatives more easily, participants in our study were sceptical regarding whether including relatives in video-based consultations was appropriate at all. In particular, they raised concerns regarding technical challenges and meeting relatives’ emotional needs via remote formats. Thus, those who would arguably benefit most from additional opportunities to take part in ethics consultations are also those whose involvement is met with the greatest barriers.

Similar concerns were raised regarding the participation of patients in remote consultations: some ethics consultants who participated in our study reported that they find it difficult to include patients in clinical ethics consultations, even in in-patient settings, and that they perceive patient involvement in remote formats as even more challenging. These findings resonate with the broader debate on patient involvement in ethics consultations [33, 34]. While AEM guidelines recommend patient participation [5], research indicates that many ethics consultations in Germany do not involve patients [35]. From an ethical perspective, it has been argued that the routine involvement of patients in ethics consultations could cause harm, as this may lead to “needless fears, unjustified expectations or false hope” [6] for patients. However, simply assuming that patients (or their relatives) cannot be included in consultations because of emotional or technical challenges appears to be based on paternalistic assumptions rather than on the attitudes of patients and relatives themselves. Hence, more research on the perspectives of these groups on ethics consultations in general [34], and digital formats in particular, is needed to identify whether these assumed harms align with the perceptions and experiences of these groups.

When this is possible without causing harm, efforts should be made to include patients in clinical ethics consultations, as their perspectives are often essential to fully understand ethical conflicts [36, 37]. It has been argued that the exclusion of patients’ perspectives can reproduce problematic power imbalances and contribute to epistemic injustice in healthcare [38]. Hence, it is important to ensure that the use of videoconferencing for ethics consultations does not lead to the automatic exclusion of patients due to concerns about feasibility. Both in in-person as well as in remote contexts, strategies to include patients should be identified and incorporated in training for ethics consultants.

### The role of emotions in online communication

Study participants highlighted the limited possibility of conveying non-verbal cues and expressing emotions in video-based ethics consultations, which is in line with findings from other healthcare settings in which video-based consultations have been implemented [23, 32, 39, 40]. Participants had diverging attitudes on how this impacted ethics consultations. Some found the equalising and structuring effects of video conferencing beneficial. They understood the limited expression of emotions in video consultations as a benefit in itself, as this could prevent specific participants from dominating the discussion. Others instead found the reduced expression of emotions disadvantageous, stating that this makes it difficult to involve relatives and patients, and makes remote formats less appropriate for emotionally charged, sensitive topics.

Generally, the sensitive topics discussed in ethics consultations can lead to intense emotions such as irritation, anger, sadness, and grief for participants [41]. Prior research demonstrates that some ethics consultants regard strong emotions as potentially distracting from the moral question and hindering ethical decision-making – both for ethics consultants themselves [42] as well as for participants [41]. Some ethics consultants may encourage participants to distance themselves from strong emotions when they view them as hindering thorough reflection or clouding thought processes [41]. Other ethics consultants, however, understand emotions as crucial for informing moral decision-making [43] and regard them as a potential source of new insights, which makes acknowledging them essential [41]. Research also indicates that many healthcare professionals regard the opportunity to share difficult emotions and receive emotional support as one of the most important benefits of ethics consultations [44].

How the role of emotions in ethics consultations is perceived – in general, as well as in video-based consultations in particular –, might hinge on how ethics consultants understand their own role and the goals they believe ethics consultation should achieve. Haltaufderheide et al. [45] distinguish two contrasting ways in which the role of ethics consultants can be understood. As part of an “analytical role”, ethics consultants act as moral problem solvers that identify, analyse and resolve ethical issues [46] by applying their knowledge to a case, explaining and clarifying normative concepts. Associated goals are to improve participants’ ability to identify ethical issues and improve patient outcomes. As part of a “hermeneutic role”, on the other hand, ethics consultants act as observers and facilitators [47] that improve and enable effective communication between participants, help clarify individual moral perspectives, and communicate support and empathy to participants [48]. As part of the latter role, ethicists can sometimes act as “conflict managers” that facilitate conversations between healthcare professionals, patients, and relatives. For many of the mentioned tasks that are part of the “hermeneutical role”, perceiving emotions and demonstrating empathy are central skills [42].

Hence, it could be hypothesised that if ethics consultants primarily understand their role as an “analytical” one with the goal of contributing to an ethically well-founded decision, they could be more likely to prefer digital formats that allow for structured discussions. In contrast, a more “hermeneutical” role that requires perceiving emotions may be more difficult to realise in video-based consultations that do not allow for the full expression of non-verbal cues and emotions as well as physical touch, and lack opportunities for informal debriefing after sessions. This explains why some participants did not regard remote consultations as appropriate for certain emotionally charged topics. Future research, as well as training on ethics consultation – particularly in remote settings – should take these different perspectives on the role of emotions, as well as the goal of ethics consultation more broadly, into account. This can help ethics consultants gain a better understanding of their own role, and how this role can be realised within remote settings.

### Strengths and limitations

To our knowledge, this is the first qualitative study on ethics consultants’ experiences with and attitudes towards remote consultation formats – particularly video-based consultations. Nonetheless, several limitations should be acknowledged. Due to its qualitative nature and small sample size, the findings of our study do not aim to be representative of the attitudes of all ethics consultants in Germany. However, our study included the perspectives of ethics consultants working in a range of different contexts and geographical areas.

The study results may be biased towards positive experiences with remote formats, given that participants with more positive attitudes may have been more likely to participate. Given the focus group format, it is possible that the views of participants with more senior positions may have influenced the discussion more strongly. Additionally, the study does not include the perspectives of patients and relatives on remote formats, whose views are critical to evaluating the acceptability of remote ethics consultation. Future research should therefore include these important perspectives and examine how remote formats affect outcomes, satisfaction, and ethical decision-making processes from the perspectives of different stakeholders.

## Conclusion

This study provides important insights on ethics consultants’ attitudes towards and experiences with remote formats, particularly video-based consultations. Our findings indicate that ethics consultants regard video-based ethics consultations as a feasible alternative when in-person meetings are not possible. The study highlights that ethics consultants have developed a range of strategies to effectively employ remote consultations, for example specific measures for preparation, follow-up, and managing emotional dynamics remotely. This knowledge could be integrated into future training courses on remote ethics consultations. In addition, our results demonstrate that further research on the effective implementation of remote ethics consultation is necessary to realise their advantages while mitigating possible disadvantages. Several areas warrant further investigation, including the relationship between experience and attitudes towards remote formats, infrastructural barriers to equitable access, and the role of emotions and professional self-understandings in remote ethics consultations. Another important question is to what extent, and how, patients and relatives can be involved in remote consultations, which requires further research on the perspectives of these groups.

## Supplementary Information

Supplementary Material 1

## Data Availability

The data generated during the current study are not publicly available to protect the privacy of the participants but are available from the corresponding author on reasonable request.
