# Supplementary material for: Experiences with remote ethics consultation: a qualitative study with ethics consultants in Germany
Source: BMC Med Ethics. 2026 Feb 10;27:40. doi: 10.1186/s12910-026-01401-x (PMC12930741; doi:10.1186/s12910-026-01401-x)
Supplement: Supplementary file 1 — Supplementary Material 1 [file 12910_2026_1401_MOESM1_ESM.docx]

**Supplement 1**

**Discussion guide for focus groups**

**Outpatient clinical ethics services**

- What experience have you made with setting up outpatient clinical ethics services?
  - What characterises outpatient clinical ethics services, how do they differ from clinical ethics services?
- What difficulties have you encountered in setting up outpatient clinical ethics services?
- What strategies were helpful in successfully implementing outpatient clinical ethics services?
- What would be necessary for the successful implementation of outpatient clinical ethics services?

**Remote ethics consultation**

- Which experiences have you made with setting up remote ethics consultation services? Which formats do you use, e.g. video conferencing, telephone, etc.?
- Which experiences have you made with setting up remote ethics consultation services in *clinical* settings?
- Which experiences have you made with setting up remote ethics consultation services in *outpatient* settings?
- How do you experience ethics consultations via video conference? How do remote ethics consultations differ from in-person consultations?
- What is your experience with involving patients in digital ethics consultations?
- Do you think there are differences in the issues that should be discussed in the context of digital ethics counselling vs in-person?
- What difficulties have you encountered with digital ethics consultation?
- What strategies were helpful in successfully implementing digital ethics consultation?
- What do you experience as particularly helpful about digital offers?
- Are digital offers helpful to improve access to ethics consultation in rural and outpatient settings?
